# Optimization and Bioreactor Scale-Up of Cellulase Production in *Trichoderma* sp. KMF006 for Higher Yield and Performance

**DOI:** 10.3390/ijms26083731

**Published:** 2025-04-15

**Authors:** Seongwoo Myeong, Yun-Yeong Lee, Jeonghee Yun

**Affiliations:** Department of Forest Products and Biotechnology, Kookmin University, Seoul 02707, Republic of Korea; mouse9610@naver.com (S.M.); yunlee529@kookmin.ac.kr (Y.-Y.L.)

**Keywords:** cellulase production, submerged fermentation (SmF), carbon source composition, hydrodynamic conditions, bioreactor scale-up

## Abstract

This study optimized operating parameters to enhance cellulase production and evaluated scale-up feasibility in submerged fermentation (SmF) using *Trichoderma* sp. KMF006. Flask-scale experiments assessed the effects of Avicel:cellulose ratios (4:0–0:4), agitation speeds (150–210 rpm), and turbulence (baffled vs. non-baffled flasks), with optimized conditions applied to a 10 L bioreactor. A 3:1 Avicel:cellulose ratio (A3C1) significantly accelerated cellulase production, reaching peak activity 6 days earlier than Avicel alone. An agitation speed of 180 rpm was optimal, balancing enzyme activity and energy efficiency. Turbulence enhanced cellulase yields, with baffled flasks increasing EG, BGL, and CBH activities 19.9-, 6.2-, and 8.9-fold, respectively, compared to the control. Biochar further improved cellulase production but only under turbulent conditions, demonstrating a synergistic effect. At the bioreactor scale, the A3-180_Imp (A3C1, 180 rpm, impeller-induced turbulence) achieved the highest enzymatic activity (33.60 U/mL EG, 3.46 U/mL BGL, and 0.63 U/mL CBH). The filter paper unit (FPU) was 84 FPU/mL, a two-fold increase compared to the control. However, excessive turbulence at 210 rpm reduced enzyme stability, emphasizing the importance of balancing shear stress. These findings provide a systematic framework for optimizing SmF conditions, highlighting the significance of balancing hydrodynamic conditions for efficient cellulase production at an industrial scale.

## 1. Introduction

Over the past few centuries, the global population has grown rapidly, leading to a significant increase in the use of petrochemical products such as plastics and fossil fuels. This excessive consumption has caused climate change and environmental pollution to become critical international issues [1,2]. In response, lignocellulosic biomass (LCB) has garnered attention as a promising alternative resource [3]. LCB, one of the most abundant and sustainable resources available globally, is categorized into three generations: first generation (agricultural crops), second generation (forestry and agricultural residues), and third generation (microalgae and bacteria) [4,5]. The use of LCB in biorefinery processes to produce bioproducts as substitutes for fossil-based resources has been actively studied [6,7]. Prominent bioproducts include biofuels and bioplastics, both of which are projected to experience steady market growth [8,9]. Germany, France, the United States, and 30 other countries have adopted the Renewable Fuel Standard (RFS) as defined by the REN21 report, blending bioethanol and biodiesel into gasoline and diesel, respectively, for transportation fuel [10]. Additionally, the European Union introduced the Single-Use Plastic Directive (EU 2019/904) as part of its Circular Economy Action Plan, mandating the use of recycled materials and promoting bioplastics [11].

LCB consists of cellulose, hemicellulose, and lignin, which are tightly bound in a rigid structure [12]. This composition makes pretreatment and saccharification essential steps for the production of bioproducts [13,14]. Saccharification is typically performed using either acid hydrolysis or cellulase enzymes. While acid hydrolysis is fast and can produce high concentrations of sugars, it requires harsh temperature conditions (200–240 °C) and generates inhibitory byproducts such as furfural, hydroxymethylfurfural (HMF), vanillin, and formic acid. In contrast, enzymatic saccharification using cellulase is gaining attention as an environmentally friendly alternative [15,16]. Cellulase is a complex enzyme system composed of endoglucanase (EG, EC 3.2.1.4), β-glucosidase (BGL, EC 3.2.1.21), and cellobiohydrolase (CBH, EC 3.2.1.91) [17]. These enzymes are secreted by microorganisms, with fungi such as *Trichoderma* sp., *Aspergillus* sp., and *Penicillium* sp. being the primary sources for industrial production [17]. However, the high cost of cellulase production remains a significant barrier to the practical production of biofuels, highlighting the need for cost-effective production processes [9,13,18].

For efficient cellulase production, two fermentation processes are commonly employed: solid-state fermentation (SSF) and submerged fermentation (SmF) [19,20]. SSF allows for high enzyme yields as it provides environmental conditions similar to those in nature, promoting microbial growth [21]. Although SmF generally results in lower enzyme activity than SSF, particularly with lignocellulosic substrates, it remains the preferred method for industrial application due to its ease of enzyme recovery, process control, and scalability [17]. To improve cellulase productivity in SmF, previous studies have focused on optimizing parameters such as nutrient composition, pH, temperature, incubation period, and aeration [22,23,24,25]. Moreover, innovative strategies, including the integration of SSF and SmF [26,27] or the use of activity-enhancing supplements [28,29], have been explored to overcome the limitations of conventional SmF systems.

This study was built upon prior research that demonstrated the potential of cellulase production by incorporating biochar during *Trichoderma* sp. cultivation in SmF [30]. Biochar, a high-carbon material with high porosity and surface area, is known to enhance microbial growth and cellulase activity by serving as a microbial habitat [31,32,33,34,35,36]. While core cultivation parameters such as temperature and pH were previously optimized for this strain [37], the present study focused on evaluating physical and structural factors including carbon source composition, agitation speed, and turbulence efficiency, as these directly affect oxygen transfer and substrate accessibility in SmF systems. Flask-scale experiments were conducted to evaluate the effects of three factors—carbon source, agitation speed, and turbulence efficiency—on cellulase production. Protein concentration and cellulase activity were measured, and the most effective conditions were identified using ANOVA-based statistical comparisons. These conditions were then applied to a bioreactor to assess the potential for scale-up, with final enzymatic activity evaluated using the Filter Paper Unit (FPU) assay.

## 2. Results and Discussion

### 2.1. Effect of Carbon Source Composition on Cellulase Activity

The time profiles of cellulase activity under different carbon source compositions are presented in Figure S1, while the maximum enzyme activities are summarized in Table 1. EG activity did not exhibit a consistent trend across different carbon compositions. In the control condition (A4C0), EG activity reached its maximum activity at 18 days (24.19 U/mL), whereas the experimental groups exhibited faster enzyme production, with the maximum activities observed between 12 and 15 days. Among these, A3C1 exhibited the highest EG activity, reaching 27.84 U/mL at 12 days. BGL and CBH exhibited more consistent patterns regardless of carbon composition. BGL activity reached its peak between 15 and 18 days, while CBH reached its maximum value between 12 and 15 days. Although no statistically significant differences were observed (*p* > 0.05), A3C1 exhibited the highest activity across all three enzymes, with the maximum values of 27.84 U/mL (EG), 0.87 U/mL (BGL), and 0.25 U/mL (CBH). Protein concentration followed a similar trend, peaking between 12 and 15 days within the range of 0.58 to 0.73 mg/mL, with no significant differences among experimental groups (*p* > 0.05).

**Figure 1:**
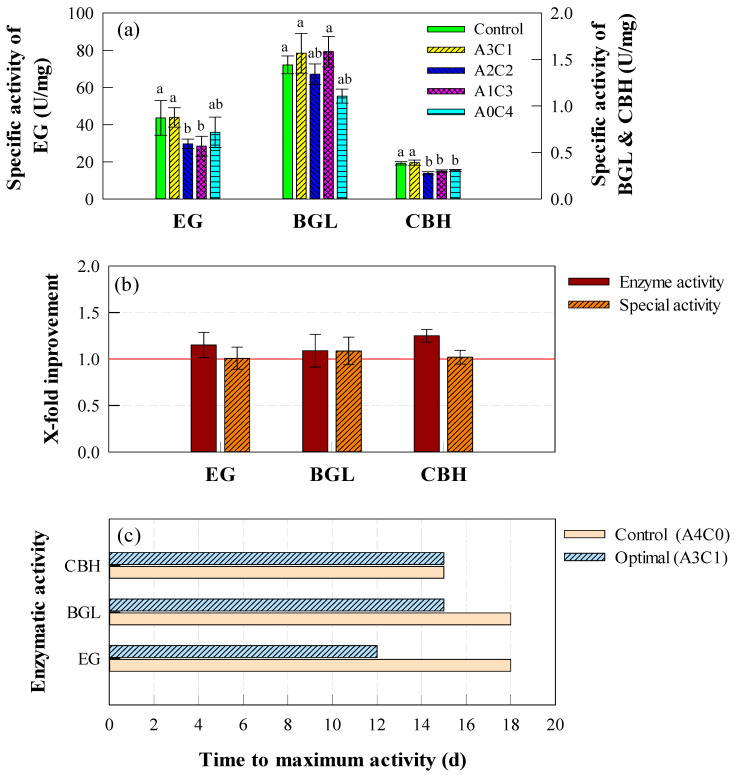
Comparison of enzymatic activities and performance under different carbon source compositions in flask-scale experiments. (**a**) Maximum specific activities (U/mg) of EG, BGL, and CBH across all experimental conditions. (**b**) X-fold improvement in enzymatic activities of cellulase under optimal (A3C1) compared to control (A4C0). X-fold values were calculated separately for enzyme activity (EA, U/mL) and specific activity (SA, U/mL). (**c**) Time to maximum enzymatic activities (days) of cellulase under control (A4C0) and optimal (A3C1) conditions. Error bars represent standard deviation (SD) from six measurements (*n* = 6). Statistical significance was determined by one-way ANOVA followed by Tukey’s HSD test (*p* < 0.05).

To further evaluate enzyme production efficiency, the maximum specific activities of EG, BGL, and CBH were analyzed (Figure 1a). Specific activity is defined as enzymatic activity normalized to protein concentration (U/mg), which allows for an evaluation of enzymatic efficiency regardless of total protein content [30]. The results showed that EG exhibited the highest specific activity in both A3C1 (43.94 U/mg) and the control (43.62 U/mg), with no significant difference between them (*p* > 0.05). The specific activities of BGL in A3C1 (1.57 U/mg) and A1C3 (1.59 U/mg) were slightly higher than those in other groups, though the differences were not significant (*p* > 0.05). In contrast, CBH activity was significantly higher in A3C1 (0.39 U/mg) and the control (0.38 U/mg) than in other groups (*p* < 0.05).

To assess the relative improvement in cellulase activity under A3C1 compared to the control, Figure 1b presents the fold increase in enzymatic activity and specific activity. Enzymatic activity in A3C1 was 1.09–1.25 times higher than in the control, while specific activity showed a 1.01–1.09-fold increase. Although these differences were relatively minor, the time required to reach the maximum activity was significantly shorter in A3C1 than in the control (Figure 1c). EG activity peaked at 12 days in A3C1, compared to 18 days in the control, while BGL activity peaked at 15 days in A3C1 and 18 days in the control. CBH showed no difference in peak timing between the two conditions.

These results suggest that, while the overall improvement in enzymatic activity was moderate, the A3C1 condition significantly reduced the cultivation time required to achieve the maximum cellulase activity. Optimizing the carbon source ratio to Avicel:cellulose = 3:1 (A3C1) was effective in accelerating enzyme production, making it the most suitable composition for cellulase production.

Avicel is widely recognized as a primary carbon source for inducing cellulase production in *Trichoderma* species [38]. As a form of microcrystalline cellulose, Avicel is relatively more hydrolysable than other insoluble cellulose substrates [38]. Several studies have demonstrated its effectiveness in cellulase production across various fungal species and fermentation systems. For instance, Silva et al. (2020 compared the cellulase activity of *Trichoderma reesei* RP698 using 12 different carbon sources, including Avicel, filter paper, and carboxymethyl cellulose (CMC), with Avicel resulting in the highest cellulase activity [39]. SmF using Avicel as the primary carbon source has successfully supported cellulase production in *Orpinomyces* sp. [40]. Similarly, *Daldinia* sp. cultivated in SmF with Avicel as the carbon source exhibited significant cellulase activity [41]. In contrast, the present study demonstrates that a mixed carbon source composed of Avicel and cellulose at a 3:1 ratio (A3C1) significantly shortened the time required to reach the maximum cellulase activity *Trichoderma* sp. KMF006, compared to Avicel alone (A4C0). This suggests that mixed substrates may facilitate a more rapid onset of enzymatic activity by influencing the induction process for cellulase production. While studies on mixed carbon sources remain limited, similar effects have been observed in previous research. Singhania et al. (2015) reported that combining wheat bran and Avicel in a 4:1 ratio enhanced cellulase production [42]. Similarly, Saini et al. (2015) demonstrated through response surface methodology (RSM) that a mixture of Avicel (0.5%, *w*/*v*), wheat bran (2.5%, *w*/*v*), and ammonium sulfate (0.53%, *w*/*v*) yielded an estimated maximum cellulase activity of 1.26 FPU/mL [43]. These findings collectively indicate that optimizing carbon source composition can be a useful strategy for improving cellulase production efficiency.

Cellulases produced by *Trichoderma* species are classified as adaptive enzymes, the expression of which is significantly induced in the presence of specific inducers [44,45]. Previous studies have suggested that insoluble cellulose alone does not effectively induce early-stage cellulase production. Instead, intermediate metabolites, particularly cello-oligosaccharides such as cellobiose, play a crucial role in triggering cellulase synthesis [46,47]. Notably, BGL functions not only as a hydrolytic enzyme but also as a transglycosylation catalyst, synthesizing β-disaccharides that serve as potent inducers of cellulase biosynthesis [44,46]. Consistent with this finding, Li et al. (2016) demonstrated that cellulase activity in *T. reesei* was significantly enhanced in the presence of β-disaccharides [45]. In this study, the use of a mixed carbon source (A3C1) shortened the time required to reach maximum enzymatic activity. EG achieved its highest activity 6 days earlier, and BGL reached its maximum activity 4 days earlier compared to the Avicel-only condition (A4C0). The earlier activation of EG likely facilitated the production of more cello-oligosaccharides, such as cellobiose, which could have contributed to the accelerated activation of BGL. Previous studies have reported that BGL production in *T. reesei* not only hydrolyzes cello-oligosaccharides but also enhances induction signaling through transglycosylation [46,47]. However, since β-disaccharides formation was not directly quantified in this study, further investigation is required to validate this hypothesis.

In conclusion, the mixed carbon source (A3C1) improved cellulase production efficiency by reducing the time required for enzyme activation in *Trichoderma* sp. KMF006 compared to Avicel alone. This reduction in activation time has potential industrial relevance, as it may enhance process efficiency in cellulase production. Since β-disaccharide formation was not directly assessed, further research is needed to quantitatively determine the role of mixed carbon sources in the early induction of cellulase biosynthesis. Therefore, future studies should explore the mechanistic relationship between early-stage induction signals, β-disaccharide accumulation, and the accelerated activation of cellulase enzymes.

### 2.2. Effect of Agitation Speed on Cellulase Activity

The influence of agitation speed on cellulase activity was examined by comparing enzyme production at 150 rpm (control), 180 rpm, and 210 rpm (Figure S2). The maximum enzyme activities under these conditions are summarized in Table 2. Overall, increasing agitation speed significantly enhanced both enzymatic activity and protein concentration, with 180 and 210 rpm yielding higher values than the control (*p* < 0.05). The maximum activities of EG, BGL, and CBH were recorded at day 15 for 180 and 210 rpm, reaching 32.04–32.35 U/mL, 3.60–3.83 U/mL, and 0.53–0.59 U/mL, respectively. In contrast, the control exhibited significantly lower maximum activities, with EG, BGL, and CBH peaking at 16.90, 1.77, and 0.33 U/mL at day 18. A similar trend was observed for protein concentration, where all conditions exhibited maximum levels at day 18. However, the control resulted in a maximum protein concentration of only 0.71 mg/mL, which was significantly lower than the 1.05–1.10 mg/mL observed at 180 and 210 rpm (*p* < 0.05). These results suggest that increasing agitation speed not only enhances enzymatic activity but also improves overall protein production efficiency.

**Figure 2:**
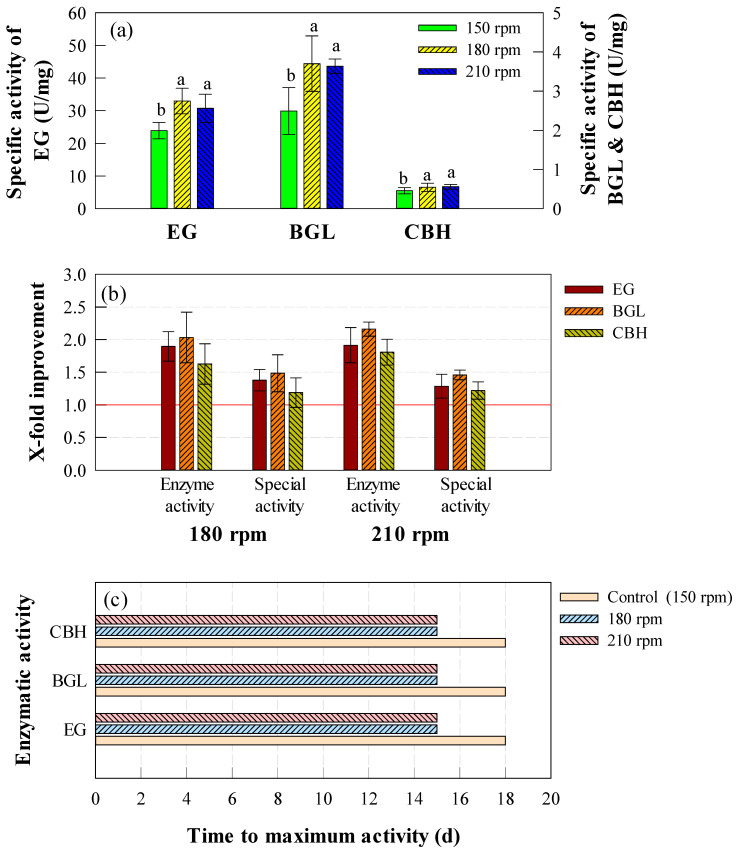
Comparison of enzymatic activities and performance under different agitation speed in flask-scale experiments. (**a**) Maximum specific activities (U/mg) of EG, BGL, and CBH across all experimental conditions. (**b**) X-fold improvement in enzymatic activities (EA, U/mL) and specific activities (SA, U/mg) of cellulase at 180 and 210 rpm compared to control (150 rpm). (**c**) Time to maximum enzymatic activities (days) of cellulase under control (150 rpm) and experimental conditions (180 and 210 rpm). Error bars represent standard deviation (SD) from six measurements (*n* = 6). Statistical significance was determined by one-way ANOVA followed by Tukey’s HSD test (*p* < 0.05).

To further evaluate the impact of agitation speed, specific enzymatic activity was analyzed (Figure 2a). Both 180 and 210 rpm exhibited significantly higher specific activities compared to the control (*p* < 0.05). The specific activities of EG, BGL, and CBH in the control were 23.89, 2.49, and 0.46 U/mg, respectively, whereas 180 and 210 rpm resulted in values ranging from 30.73 to 32.95 U/mg (EG), 3.64 to 3.70 U/mg (BGL), and 0.54 to 0.56 U/mg (CBH). Notably, there was no significant difference between 180 and 210 rpm (*p* > 0.05), indicating that further increasing agitation speed beyond 180 rpm does not provide additional enzymatic efficiency benefits. This may suggest that the physiological limits of the strain were reached at 180 rpm, beyond which additional agitation did not further stimulate enzyme synthesis [48].

**Figure 3:**
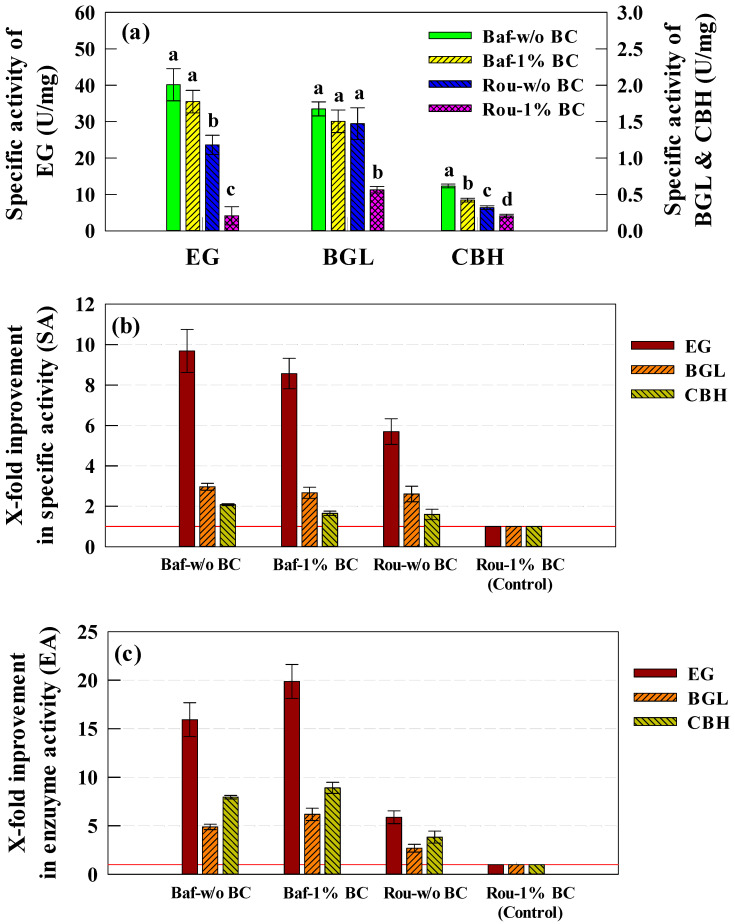
Comparison of enzymatic activities and x-fold improvements under different turbulence conditions in flask-scale experiments. (**a**) Maximum specific activities (U/mg) of EG, BGL, and CBH across four experimental conditions. (**b**) X-fold improvement in specific activities (SA, U/mL) of cellulase under turbulence conditions relative to Rou-1% BC as the control. (**c**) X-fold improvement in enzymatic activities (EA, U/mL) of cellulase under turbulence conditions relative to Rou-1% BC as the control. Error bars represent standard deviation (SD) from six measurements (*n* = 6). Statistical significance was determined by one-way ANOVA followed by Tukey’s HSD test (*p* < 0.05).

The relative enhancement of cellulase activity at higher agitation speeds compared to the control is illustrated in Figure 2b. At 180 and 210 rpm, the maximum activity increased by 1.90–1.91-fold for EG, 2.03–2.16-fold for BGL, and 1.63–1.81-fold for CBH compared to the control. A similar pattern was observed in specific activity, where EG, BGL, and CBH exhibited 1.29–1.38-, 1.46–1.49-, and 1.19–1.22-fold improvements, respectively. In addition to enhanced activity, higher agitation speeds also accelerated enzyme production as shown in Figure 3b. While the control required 18 days to reach peak cellulase activity, cultures at 180 and 210 rpm reached their maximum enzymatic activity on day 15, demonstrating the role of agitation in expediting enzyme biosynthesis.

Although no statistically significant difference was observed between 180 and 210 rpm, increasing agitation speed above 180 rpm did not result in additional enzymatic improvements. In addition, considering the potential increase in energy consumption at higher agitation speeds, 180 rpm is suggested as the optimal condition for cellulase production.

Agitation speed is a key parameter influencing cellulase production by regulating oxygen transfer, shear stress, and fungal morphology. Increased agitation enhances turbulent flow, thereby improving oxygen transfer efficiency, which is crucial for microbial metabolism and enzyme secretion [48]. However, excessive shear stress at high agitation speeds may cause mechanical damage to fungal hyphae, whereas insufficient agitation can lead to pellet formation, restricting oxygen and nutrient diffusion [17,48]. Several studies have evaluated the effect of agitation speed on cellulase production. Sirohi et al. (2019) evaluated the optimal agitation speed for cellulase production in *Trichoderma* sp. using response surface methodology (RSM) [49]. Pea hulls were used as the substrate and the cultivation temperature was set at 30 °C. Agitation speed was tested within a range of 93 to 127 rpm, and RSM analysis identified 120 rpm as the optimal condition. In another study using *Pycnoporus* sp., cellulase production was assessed under varying agitation speeds [50]. The strain was cultivated at 30 °C using palm oil mill effluent and oil palm frond as substrates. The results indicated that the highest cellulase activity was achieved at 350 rpm among tested speeds ranging from 100 to 500 rpm. In bacterial systems, agitation speed also plays a crucial role. A study on *Micrococcus* sp. investigated the effect of agitation speed on cellulase production using carboxymethyl cellulose (CMC) as the substrate. The strain was incubated at 25 °C, and experimental results showed that maximum cellulase production occurred at 50 rpm when tested across a range of 0 to 200 rpm [51]. Direct comparisons among these studies are challenging due to differences in microbial strains, cultivation conditions, and experimental design. Nevertheless, these findings collectively highlight the critical role of agitation speed in optimizing cellulase production and emphasize the need for strain-specific optimization to achieve maximal enzyme yields.

### 2.3. Effect of Turbulence on Cellulase Activity

The impact of turbulence on cellulase activity was assessed by comparing baffled and non-baffled conditions with or without 1% biochar supplementation. The time profiles of enzymatic activity under these conditions are presented in Figure S3, and the maximum enzyme activities are summarized in Table 2. Overall, the presence of turbulence significantly enhanced cellulase activity, while biochar alone did not consistently improve enzyme production unless turbulence was present. The maximum enzymatic activity values confirm this trend (Table 2), with Baf-1% BC exhibiting the highest enzyme production, reaching 34.41 U/mL (EG), 1.46 U/mL (BGL), and 0.33 U/mL (CBH) at the peak point. The Baf-w/o BC condition also demonstrated increased activity, with values of 27.59, 1.15, and 0.30 U/mL, respectively. In contrast, non-baffled conditions resulted in significantly lower cellulase production, with Rou-w/o BC exhibiting higher activity than Rou-1% BC.

To further analyze enzyme production efficiency, specific enzymatic activity was evaluated by normalizing enzymatic activity to protein concentration (Figure 3a). In contrast to total enzymatic activity, specific activity was not significantly affected by biochar addition under turbulent conditions (*p* > 0.05). EG exhibited high specific activity in all turbulent conditions, reaching 35.48–40.14 U/mg, irrespective of biochar supplementation. There was no significant difference between Baf-w/o BC and Baf-1% BC (*p* > 0.05), but both exhibited significantly higher specific activity than non-baffled conditions (*p* < 0.05). For BGL, specific activity ranged from 1.50 to 1.68 U/mg, with no significant differences between Baf-w/o BC and Baf-1% BC. However, Rou-1% BC showed the lowest specific activity, significantly lower than all other conditions (*p* < 0.05). For CBH, all experimental groups exhibited significant differences (*p* < 0.05), with Baf-w/o BC showing the highest specific activity at 0.62 U/mg, followed by Baf-1% BC (0.42 U/mg), Rou-w/o BC (0.32 U/mg), and Rou-1% BC (0.21 U/mg). These results indicate that turbulence significantly improved specific enzymatic activity for EG and CBH, whereas biochar supplementation had a greater impact on BGL-specific activity.

To quantify the improvement in specific activity relative to the control, Rou-1% BC was set as the baseline condition (Figure 3b). Baf-w/o exhibited the highest fold-change improvement in specific activity, increasing EG, BGL, and CBH activity by 9.7-, 3.0-, and 2.1-fold, respectively. In comparison, Baf-1% BC showed slightly lower increases (8.6-, 2.7-, and 1.7-fold, respectively), while Rou-w/o BC exhibited moderate improvements (5.7-, 2.6-, and 1.6-fold). These findings indicate that turbulence alone plays a crucial role in enhancing specific cellulase activity, whereas biochar supplementation does not significantly alter specific enzyme efficiency.

However, specific activity alone does not provide a complete picture of enzyme production efficiency. Therefore, total enzymatic activity was analyzed to evaluate the overall cellulase production yield (Figure 3c). Compared to the control (Rou-1% BC), Baf-1% BC exhibited the highest fold-change improvement in total enzymatic activity, with EG, BGL, and CBH increasing by 19.9-, 6.2-, and 8.9-fold, respectively. Baf-w/o BC also demonstrated substantial increases (15.9-, 4.9-, and 8.0-fold), confirming that turbulence alone significantly enhances enzyme production. Interestingly, in non-baffled conditions, Rou-w/o BC showed moderate improvements (5.9-, 2.7-, and 3.8-fold), whereas Rou-1% BC (control) exhibited the lowest values.

When biochar was introduced under turbulent conditions, Baf-1% BC exhibited the highest total enzyme yield despite having slightly lower specific activity than Baf-w/o BC. This discrepancy is attributed to the higher protein concentration observed in Baf-1% BC, which resulted in an overall increase in cellulase production. Thus, although Baf-w/o BC showed the highest specific activity, Baf-1% BC was the optimal condition for cellulase production, as it maximized both enzymatic activity and total yield. These results highlight the synergistic effect of turbulence and biochar, where biochar supplementation enhances cellulase production only when combined with adequate turbulence.

Biochar has been reported to enhance microbial growth and enzyme activity due to its highly porous structure, which offers a favorable microenvironment and serves as a source of readily available carbon and nitrogen [30,36]. Our previous study investigated the effect of biochar supplementation on cellulase activity using the same *Trichoderma* sp. KMF006 strain under baffled flask conditions [30]. In that study, biochar addition led to a 12.1-, 5.8-, and 7.2-fold increase in EG, BGL, and CBH activity, respectively. The current study, which employed the same strain and baffled flasks to promote turbulence, demonstrated a similar positive trend (Table 1). Enzyme activities were significantly higher in biochar-supplemented groups, reaffirming its stimulatory effect on cellulase production. However, this enhancement was observed only under turbulent conditions. In non-baffled flasks, biochar supplementation did not improve cellulase activity and even led to lower values in some cases. This indicates that the effectiveness of biochar may depend on specific hydrodynamic conditions, particularly the intensity of turbulence. Since biochar’s stimulatory effect is closely related to its interaction with fungal growth and reproduction [36], sufficient turbulence may help maintain particle suspension and enhance microbial contact. In contrast, low turbulence may lead to particle sedimentation or aggregation, thereby reducing its effectiveness. Although the exact mechanism remains unclear, further studies are needed to clarify how hydrodynamic conditions influence biochar behavior in submerged systems.

Although agitation can introduce turbulence, its effect becomes increasingly limited when scaling up to larger bioreactors, where maintaining efficient oxygen transfer and mixing is more challenging [52]. Thus, optimizing turbulence conditions is crucial for improving cellulase production beyond what agitation alone can achieve. The strong influence of turbulence has been demonstrated in several studies, where optimized turbulent flow increased cellulase production [53,54]. The effect of turbulence on cellulase production was evaluated in *Pichia pastoris* by comparing baffled and non-baffled flasks [54]. In baffled flasks, turbulence-induced aeration enhanced protein synthesis and enzyme activity. Mitra et al. (2011) demonstrated that turbulence improved oxygen and nutrient distribution, leading to a significant increase in FPase activity in *Chaetomium crispatum* [53]. The turbulence effect can be attributed to enhanced oxygen transfer and improved mixing dynamics, both of which are critical for cellulase synthesis in filamentous fungal cultures, such as *Trichoderma* species [55]. In submerged fermentation, filamentous fungal growth increases broth viscosity, which can exacerbate oxygen transfer limitations and hinder enzyme production. Turbulence mitigates this issue by generating shear stress, which reduces viscosity and decreases bubble size, thereby increasing the gas–liquid interfacial area and enhancing oxygen solubility [53]. This is particularly important for *Trichoderma* sp., as its cellulase production is highly sensitive to oxygen availability [52]. Beyond oxygen transfer, turbulence also plays a key role in maintaining a stable microenvironment within the fermentation broth. Efficient mixing prevents the accumulation of extracellular metabolic byproducts, which could otherwise inhibit enzyme production [56]. Baffles, in particular, generate strong shear forces that improve the homogeneity of nutrient and oxygen distribution, creating favorable conditions for cellulase synthesis [55]. However, *Trichoderma* cellulase activity is highly sensitive to shear stress, making it essential to balance turbulence levels. While moderate turbulence enhances enzyme production, excessive shear stress can damage fungal hyphae, leading to reduced cellulase productivity [55]. This highlights the importance of optimizing turbulence conditions to maximize cellulase production while minimizing the potential negative effects on fungal integrity.

### 2.4. Scale-Up of Cellulase Production in a SmF Bioreactor

The SmF reactor experiment was conducted to evaluate the effects of scaling up the optimal flask-level culture conditions. The basic control was set as C0-150 (Avicel:Cellulose = 4:0, 150 rpm), and the optimal conditions from the flask experiments were sequentially applied, including carbon source composition (A3C1), agitation speed (180 and 210 rpm), and turbulence formation using an impeller.

Figure S4 presents the time profiles of enzymatic activity and protein concentration in the SmF reactor, which were monitored for up to 12 days. In contrast to the flask-scale experiments, where batch aeration allowed prolonged enzyme production for up to 18 days, the continuous aeration system in the reactor led to a shorter enzyme activity maintenance period. Furthermore, although our previous study monitored enzymatic activities for 18 days, it decreased after day 12 in the reactor system. Additionally, considering the constraints of subsequent saccharification processes, prolonged monitoring beyond 12 days was deemed impractical. Excluding the control, enzymatic activity and protein concentration continuously increased until day 12 across all experimental groups. Among them, A3-180_Imp exhibited the highest final enzymatic activities. In contrast, C0-150 displayed the lowest enzymatic activities, and its protein concentration did not significantly increase after day 9.

**Figure 4:**
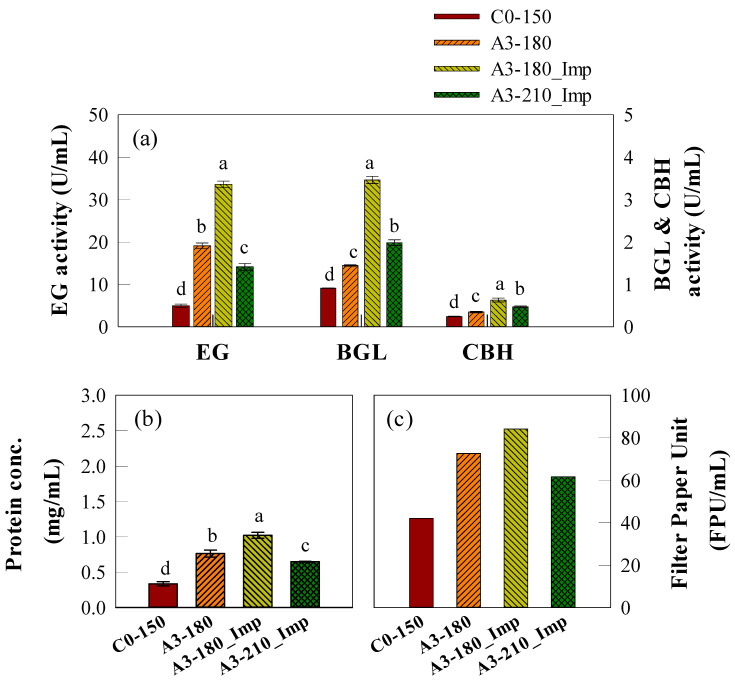
Final enzymatic activities (EG, BGL, and CBH) and filter paper unit (FPU) measurements of KMF006 cultured in a SmF reactor. (**a**) Enzymatic activities of EG, BGL, and CBH. (**b**) Protein concentrations of each experimental group. (**c**) FPU measurements representing overall cellulase activity. Experimental conditions are represented as follows: C0-150, Control condition (A4C0, 150 rpm); A3-180, A3C1, 180 rpm; A3-180_Imp, A3C1, 180 rpm with impeller adjustment; A3-210_Imp, A3C1, 210 rpm with impeller adjustment. Error bars represent standard deviation (SD) from three biological replicates measured in duplicate (*n* = 6). Statistical significance was determined by one-way ANOVA followed by Tukey’s HSD test (*p* < 0.05).

The maximum enzymatic activities at the final time point are shown in Figure 4a. The control reactor (C0-150), operating under the most basic conditions, exhibited the lowest enzymatic activities for all cellulase components, with EG, BGL, and CBH values of 4.99, 0.91, and 0.27 U/mL, respectively. When the optimal carbon source composition (A3C1) and agitation speed (180 rpm) were applied, these significantly increased to 19.12, 1.45, and 0.35 U/mL, respectively, in the A3-180 reactor. Further enhancement was observed when turbulence was introduced using an impeller (A3-180_Imp), leading to the highest activities. Among all tested conditions, A3-180_Imp exhibited the highest enzyme activities, reaching 33.60, 3.46, and 0.63 U/mL for EG, BGL, and CBH, respectively. In contrast, when the agitation speed was increased to 210 rpm (A3-210_Imp), the synergistic effect with turbulence was not observed. Instead, enzymatic activity decreased to 14.15, 1.99, and 0.48 U/mL, showing lower values than A3-180 despite the presence of turbulence. Particularly for EG, enzymatic activity in A3-210_Imp was significantly lower than in A3-180, indicating that excessive agitation speed negatively affected enzyme production.

Protein concentration trends exhibited a similar pattern to enzymatic activities, with the highest values observed in A3-180_Imp (1.02 mg/mL), followed by A3-180 (0.76 mg/mL), A3-210_Imp (0.65 mg/mL), and C0-150 (0.34 mg/mL). These results suggest that, at the reactor scale, an agitation speed of 180 rpm was optimal for cellulase production, and excessive agitation and turbulence may have had a detrimental effect on enzyme stability or microbial growth, potentially leading to reduced enzyme secretion.

Figure 4c illustrates the filter paper unit (FPU) per mL, which represents overall cellulase activity. FPU trends aligned with those of enzymatic activity and protein concentration. The highest FPU was observed in A3-180_Imp (84 FPU/mL), followed by A3-180 (72 FPU/mL) and A3-210_Imp (61 FPU/mL). The control reactor (C0-150) exhibited the lowest FPU value at 42 FPU/mL. Since the FPU reflects the total cellulase activity available for saccharification, these findings indicate that optimization at the reactor scale can have a direct impact on the efficiency of subsequent hydrolysis processes.

SmF, a liquid-phase cultivation system, provides a uniform growth environment and allows precise process control, making it widely utilized for the industrial production of cellulase [57]. The efficiency of cellulase production in SmF is influenced by various operating parameters (such as temperature, pH, carbon source composition, aeration, and turbulence), and optimizing these variables has been a key strategy for enhancing enzyme production [57,58]. Turbulence, in particular, plays a crucial role in improving oxygen transfer and mass distribution, ultimately affecting fungal metabolism and enzyme secretion [59]. Accordingly, various bioreactor designs have been introduced and evaluated to enhance turbulence effects and optimize process performance. Of these, stirred tank reactors (STRs) generate turbulence through mechanical agitation to enhance oxygen transfer and mixing. They also allow precise control over key process parameters, such as temperature and pH, making them widely used in industrial applications [57,58]. Airlift reactors, on the other hand, utilize natural gas flow to induce turbulence and optimize oxygen transfer while minimizing shear stress, thereby preventing hyphal damage and simultaneously enhancing enzyme productivity [58]. The impact of turbulence control on cellulase production has been experimentally validated in several studies. In STR, an optimized agitation speed of 220 rpm and an aeration rate of 0.6 vvm led to high cellulase activity when cultivating *Penicillium funiculosum* [60]. A comparative study between STRs and airlift reactors demonstrated that STRs achieved superior cellulase activity [61]. Meanwhile, applying mechanical agitation in a 35 L airlift bioreactor successfully promoted fungal growth and cellulase production [62]. Further supporting this, turbulence generated through mechanical agitation was found to significantly improve oxygen transfer efficiency in airlift bioreactors [59].

To further investigate the role of turbulence control in SmF, this study systematically examined how turbulence affects cellulase activity by sequentially applying optimized conditions from flask experiments to a reactor system. Among the tested conditions, the highest cellulase activity was observed under the A3-180_Imp condition (A3C1, 180 rpm, and impeller-attached for turbulence). In contrast, increasing agitation speed to 210 rpm led to a decrease in cellulase activity (Figure 4). These findings suggest that while turbulence enhances oxygen transfer and nutrient distribution, excessive agitation may generate shear stress that negatively impacts fungal morphology and enzyme stability [55]. Previous studies have demonstrated that high agitation rates reduce cellulase yield, likely due to shear stress affecting fungal structure and enzymatic stability [63,64]. Consistent with previous studies, this study experimentally verified that turbulence regulation plays a critical role in cellulase production in SmF. Since optimal conditions may vary depending on the experimental environment and fungal strain used, direct comparisons with previous studies remain challenging. Nevertheless, these findings emphasize that the precise control of agitation speed and turbulence levels in SmF systems can be an effective strategy for maximizing cellulase production efficiency.

## 3. Materials and Methods

### 3.1. Microorganism and Pre-Culture Conditions

A cellulase-producing strain, *Trichoderma* sp. KMF006, which was previously isolated [37], was used in this study. The strain was cultivated on malt extract agar (MEA), which consisted of 20 g·L^−1^ of malt extract, 20 g·L^−1^ of glucose, 1 g·L^−1^ of peptone, and 20 g·L^−1^ of agar. The plates were incubated at 26 °C until the surface was densely covered with green conidia [30]. The cultured strain was stored at 4 °C until use in pre-culture.

For pre-culture, strain KMF006 was grown in 250 mL baffled flasks containing 100 mL of potato dextrose broth (PDB). Circular plugs (1 cm in diameter) were cut from the MEA plates, and 10 plugs were inoculated into the PDB medium. The flasks were incubated at 26 °C with shaking at 150 rpm for 5 days, after which the pre-culture was used as the inoculum [30].

### 3.2. Assessment of Carbon Source Composition on Cellulase Production

To evaluate the optimal cellulase activity based on carbon source composition, Avicel and cellulose (Daejung Chemicals & Metals Co., Ltd., Gyeonggi-do, Republic of Korea) were used as carbon sources in various ratios (Table 2). The total carbon source concentration was fixed at 2% (*w*/*v*) in all experimental groups. The control group (A4C0) consisted of 2% (*w*/*v*) Avicel, while the experimental groups (A3C1, A2C2, A1C3, and A0C4) contained Avicel and cellulose mixtures at ratios of 3:1, 1:1, 1:3, and 0:4 (*w*/*v*). The cellulose used in this study had an average particle size of 20–100 μm.

Statistical analysis was performed using one-way ANOVA followed by Tukey’s HSD test. See Section 3.6.4 for details.

The basal medium composition was adapted from a previous study [30] and included yeast extract (10 g·L^−1^), H_2_PO_4_ (5 g·L^−1^), K_2_HPO_4_ (5 g·L^−1^), and MgSO_4_·7H_2_O (3 g·L^−1^), with the initial pH adjusted to 5.03. Pre-culture inoculum (5%, *v*/*v*) was added to each flask, and cultures were incubated at 31.3 °C with shaking at 150 rpm.

All experiments were conducted in 150 mL flasks with a working volume of 100 mL. Based on previous findings, 1% (*w*/*v*) biochar was uniformly added to all experimental groups to enhance cellulase activity [30]. The biochar was sterilized at 121 °C for 30 min prior to use. To monitor cellulase activity, 1 mL of culture medium was sampled at 3-day intervals and centrifuged at 10,000 rpm for 10 min to obtain the supernatant. All experiments were performed in triplicate to ensure reproducibility.

### 3.3. Assessment of Agitation Speed on Cellulase Production

To investigate the effect of agitation speed on cellulase activity, experiments were conducted at agitation speeds of 150 rpm (control), 180 rpm, and 210 rpm (experimental groups) as shown in Table 1. The carbon source composition was fixed at Avicel:cellulose = 3:1 (*w*/*v*), as determined in previous experiments, with a total carbon source concentration of 2% (*w*/*v*). The medium composition, culture conditions, working volume, biochar addition, and sampling procedure were identical to those described in Section 2.2. All experiments were performed in triplicate to ensure reproducibility.

### 3.4. Assessment of Turbulence Efficiency on Cellulase Production

To evaluate the effect of turbulence on cellulase activity, experiments were conducted using 250 mL baffled-bottom flasks and round-bottom flasks. Baffled-bottom flasks (Baf) were used to enhance turbulence, whereas round-bottom flasks (Rou) served as a control with reduced turbulence. Based on the results obtained from cellulase activity experiments with different carbon source compositions and agitation speeds, the carbon source ratio was fixed at A3C1 (Avicel:cellulose = 3:1, *w*/*v*), and the agitation speed was set at 180 rpm (Table 1). Each flask contained 100 mL of culture medium as the working volume.

To investigate whether the effect of turbulence on cellulase activity varied depending on biochar addition, the experimental groups were divided into four conditions (Table 1). The Baf-w/o BC group was incubated in baffled-bottom flasks without biochar, and the Baf-1% BC group was cultured in baffled-bottom flasks with 1% (*w*/*v*) biochar. In contrast, the Rou-w/o BC group was cultured in round-bottom flasks without biochar, while the Rou-1% BC group was cultured in the same type of flask but with 1% (*w*/*v*) biochar. The medium composition, culture conditions, and sampling procedures were identical to those described in Section 2.2. All experiments were performed in triplicate to ensure reproducibility, and cellulase activity was monitored at 3-day intervals.

### 3.5. SmF Bioreactor Conditions for High Activity Cellulase Production

To evaluate cellulase activity in a bioreactor system, a 10 L SmF bioreactor was employed with a working volume of 6 L. The medium composition consisted of yeast extract (10 g·L^−1^), H_2_PO_4_ (5 g·L^−1^), KH_2_PO_4_ (5 g·L^−1^), and MgSO_4_·7H_2_O (3 g·L^−1^), with the initial pH adjusted to 5.03. After inoculating 5% (*v*/*v*) pre-culture, aeration was maintained at a flow rate of 2 L/min, and the bioreactor was operated at 31.3 °C. The optimal culture conditions for carbon source composition, agitation speed, and turbulence, which were determined from flask-scale experiments, were sequentially applied to the SmF bioreactor.

Bioreactor experiments were conducted under four different operational conditions to analyze their effect on cellulase activity (Table 1). The control group (C0-150) was wetted with Avicel (2%, *w*/*v*) as the sole carbon source, agitated at 150 rpm, with no turbulence. Experimental group 1 (A3-180) applied the optimal carbon source composition (Avicel:cellulose = 3:1, *w*/*v*) and agitation speed (180 rpm) without turbulence. Experimental group 2 (A3-180_Imp) followed the same conditions as experimental group 1 but included turbulence, which was generated by attaching an impeller. Lastly, experimental group 3 (A3-210_Imp) applied a higher agitation speed (210 rpm) with turbulence to assess the combined effects of high agitation and turbulence.

Samples were collected at 3-day intervals during the culture period to monitor cellulase activity and protein concentration. After cultivation, the culture broth was subjected to filtration and concentration processes to evaluate cellulase production yield. The medium was centrifuged at 13,000 rpm for 10 min at 4 °C to separate the supernatant, which was then filtered using filter paper (Whatman No. 10, Cytiva, UK). The filtered supernatant was concentrated to 1/60 of its initial volume using an Amicon^®^ Stirred Cell (UFSC40001, Merck Millipore, Darmstadt, Germany) equipped with a 10 kDa cutoff polyethersulfone membrane (PG 10, Merck Millipore, Germany). The concentrated cellulase was evaluated for enzymatic activity using the Filter Paper Unit (FPU) assay. For each bioreactor condition, a single run was performed. During cultivation, three biological samples were collected at 3-day intervals to monitor enzymatic activity. Each sample was analyzed in duplicate, and the results are expressed as mean ± standard deviation (SD).

### 3.6. Enzymatic Activity and Protein Analysis

#### 3.6.1. Enzymatic Activity Analysis

EG activity was measured using the Somogyi–Nelson method [64]. The substrate used was 2% carboxymethylcellulose (CMC, Sigma-Aldrich, Burlington, MA, USA) dissolved in a 0.1 M sodium citrate buffer (pH 5.0). A reaction mixture consisting of 45 μL of CMC solution and 5 μL of enzyme sample was incubated at 60 °C for 30 min. After the reaction, 50 μL of Nelson reagent and 850 μL of distilled water were added to the mixture to analyze the amount of reducing sugars produced. Absorbance was measured at 650 nm, and one unit (U) of enzymatic activity (EG) was defined as the amount of enzyme required to release 1 μmol of glucose per minute under the assay conditions.

BGL and CBH activities were evaluated using 0.1 M p-nitrophenyl-β-D-glycopyranoside (pNPG, Sigma-Aldrich, USA) and 0.1 M p-nitrophenyl-β-D-cellobioside (pNPC, Sigma-Adrich, USA) as substrates, respectively. The substrates were prepared by dissolving in a 0.1 M sodium citrate buffer (pH 5.0). A reaction mixture of 20 μL of substrate and 20 μL of enzyme sample was incubated at 65 °C for 15 min. The reaction was terminated by adding 50 μL of 2 M Na_2_CO_3_, and the amount of 4-nitrophenol produced was quantified by measuring absorbance at 405 nm. One unit (U) of enzymatic activity (BGL and CBH) was defined as the amount of enzyme required to release 1 μmol of 4-nitrophenol per minute under the assay conditions [65].

All experiments were performed in duplicate, and the results were calculated as the mean values. Absorbance was measured using a UV/Vis spectrophotometer (Synergy LX multi-mode reader, Biotek, Agilent Technologies, Winooski, VT, USA).

#### 3.6.2. Protein Concentration Analysis

Protein concentration was analyzed to monitor the growth of *Trichoderma* sp. KMF006 using a protein assay kit (Bio-rad, Hercules, CA, USA) based on the Bradford’s method [66]. The assay was conducted by mixing 400 μL of distilled water, 100 μL of protein assay reagent, and 10 μL of enzyme sample, followed by incubation at room temperature for 5 min. After the reaction, the supernatant was collected, and the absorbance was measured at 595 nm using a UV/Vis spectrophotometer (Agilent Technologies, USA). Protein concentration (mg/mL) in the solution was determined using a standard curve prepared with bovine serum albumin (BSA) as the standard protein. All experiments were performed in triplicate.

#### 3.6.3. Filter Paper Unit (FPU) Analysis

FPU was measured following the NREL/TP-510-42628 (2008) protocol [67,68]. A 1 cm × 6 cm Whatman No. 1 filter paper (50 mg) was used as the substrate, which was placed in a 15 mL test tube. Subsequently, 1 mL of 0.1 M sodium citrate buffer (pH 5.0) and 0.5 mL of enzyme sample were added. The reaction mixture was incubated at 50 °C for 1 h. To terminate the reaction, 3 mL of 3,5-dinitrosalicyclic acid (DNS) reagent was added, and the tube was heated in boiling water for 5 min, followed by cooling at room temperature for 10 min. The cooled sample was centrifuged at 13,000 rpm for 10 min at 4 °C to separate the supernatant. A 40 μL aliquot of the supernatant was mixed with 500 μL of distilled water, and the absorbance was measured at 540 nm using a UV/Vis spectrophotometer (Agilent Technologies, USA).

#### 3.6.4. Statistical Analysis

For flask-scale experiments, three independent cultures (biological replicates) were prepared under each condition. Each sample was measured in duplicate (technical replicates), and the results were expressed as mean ± standard deviation (SD), with a total of *n* = 6 per condition. T-test and one-way ANOVA followed by Tukey’s HSD test for multiple comparisons were conducted using R software (v. 4.4.1). Statistical significance was considered at *p* < 0.05.

## 4. Conclusions

This study systematically evaluated the effects of carbon source composition, agitation speed, and turbulence on cellulase production by *Trichoderma* sp. KMF006 in both flask-scale and bioreactor-scale submerged fermentation (SmF) systems. The findings highlight key process parameters that influence enzyme activity and production efficiency.

Optimizing the carbon source composition to a 3:1 ratio of Avicel to cellulose (A3C1) significantly enhanced cellulase production, particularly by accelerating the time to peak enzymatic activity compared to Avicel alone. Agitation speed was also found to be a critical factor, with both 180 rpm and 210 rpm yielding similar enzymatic activity and protein concentrations. However, considering energy consumption and process efficiency, 180 rpm was determined to be the optimal condition for cellulase production. Turbulence played a significant role in enhancing cellulase production by improving oxygen transfer and nutrient distribution. In flask-scale experiments, baffled flasks resulted in higher enzymatic activity compared to non-baffled conditions, confirming the positive effect of turbulence. Additionally, biochar supplementation further enhanced enzyme production, but only in the presence of turbulence, indicating a synergistic effect.

At the reactor scale, turbulence was introduced via an impeller, which further improved cellulase production under optimized conditions. The A3-180_Imp condition (A3C1, 180 rpm, impeller-induced turbulence) yielded the highest enzymatic efficiency and protein concentration, demonstrating that precise control over agitation and turbulence is essential for large-scale cellulase production. These findings emphasize the need to balance aeration and shear stress to maximize enzyme yields in industrial SmF systems.

While this study systematically evaluated the effects of carbon source composition, agitation speed, and turbulence on cellulase production, certain aspects require further investigation. The direct quantification of intermediates involved in cellulase induction was not performed. Additionally, although turbulence was found to enhance cellulase production, its interaction with other process parameters, such as aeration rate and shear stress distribution, remains to be fully elucidated. Future studies should focus on elucidating the role of intermediates in cellulase induction, investigating the interactions between turbulence and key process parameters, and developing strategies for large-scale process optimization.

Nevertheless, this study demonstrated the significant impact of carbon source composition, agitation speed, and turbulence control in SmF, providing a comprehensive framework for optimizing cellulase production. The findings contribute to advancing cellulase bioprocessing strategies and offer practical insights for improving enzyme production efficiency in industrial applications.

## Figures and Tables

**Table 1:** The maximum enzymatic activities and protein concentration under different flask-level experimental conditions.

**C Source Comp. ^(1)^**	**EG (U/mL)**	**BGL (U/mL)**	**CBH (U/mL)**	**Protein (mg/mL)**
Control (A4C0)	24.19 ± 5.18 ^ab^	0.80 ± 0.05 ^ab^	0.20 ± 0.01 ^b^	0.58 ± 0.09 ^a^
A3C1	27.84 ± 3.29 ^ab^	0.87 ± 0.14 ^a^	0.25 ± 0.01 ^a^	0.68 ± 0.19 ^a^
A2C2	17.93 ± 5.55 ^b^	0.72 ± 0.05 ^ab^	0.18 ± 0.03 ^b^	0.73 ± 0.06 ^a^
A1C3	16.75 ± 3.13 ^b^	0.70 ± 0.07 ^b^	0.17 ± 0.02 ^b^	0.60 ± 0.07 ^a^
A0C4	21.38 ± 4.88 ^ab^	0.66 ± 0.05 ^b^	0.20 ± 0.01 ^b^	0.66 ± 0.06 ^a^
**Agitation speed ^(2)^**	**EG (U/mL)**	**BGL (U/mL)**	**CBH (U/mL)**	**Protein (mg/mL)**
150 rpm	16.90 ± 2.12 ^b^	1.77 ± 0.42 ^b^	0.33 ± 0.06 ^b^	0.71 ± 0.08 ^b^
180 rpm	32.04 ± 3.82 ^a^	3.60 ± 0.69 ^a^	0.53 ± 0.10 ^a^	1.05 ± 0.07 ^a^
210 rpm	32.35 ± 4.57 ^a^	3.83 ± 0.19 ^a^	0.59 ± 0.06 ^a^	1.10 ± 0.05 ^a^
**Turbulence effi. ^(3)^**	**EG (U/mL)**	**BGL (U/mL)**	**CBH (U/mL)**	**Protein (mg/mL)**
Baf-w/o BC	27.59 ± 3.03 ^b^	1.15 ± 0.07 ^b^	0.30 ± 0.01 ^b^	0.69 ± 0.04 ^b^
Baf-1% BC	34.41 ± 3.02 ^a^	1.46 ± 0.15 ^a^	0.33 ± 0.02 ^a^	0.97 ± 0.03 ^a^
Rou-w/o BC	10.18 ± 1.14 ^c^	0.63 ± 0.09 ^c^	0.14 ± 0.02 ^c^	0.43 ± 0.05 ^c^
Rou-1% BC	1.73 ± 1.05 ^d^	0.24 ± 0.02 ^d^	0.04 ± 0.00 ^d^	0.42 ± 0.07 ^c^

^(1)^ C source composition: Avicel (A) and cellulose (C) were mixed at different ratios (*w*/*v*), where the number represents the respective proportions. ^(2)^ Agitation speed: Fixed carbon source ratio (A3C1) was used at agitation speeds of 150, 180, and 210 rpm. ^(3)^ Turbulence efficiency: Fixed carbon source ratio (A3C1) at 180 rpm was tested in baffled and round flasks, with and without biochar (1%, *w*/*v*). Significant differences among the experimental conditions are represented by different letters (*p* < 0.05). All data are expressed as mean ± standard deviation (SD) from six measurements (*n* = 6).

**Table 2:** Experimental conditions for flask- and reactor-level experiments.

Scale	Operational parameter	Experiment	Experimental Condition
Flask	C source composition ^(1)^	A4C0 (Control)	Avicel:Cellulose = 4:0 (*w*/*v*), 150 rpm
		A3C1	Avicel:Cellulose = 3:1 (*w*/*v*), 150 rpm
		A2C2	Avicel:Cellulose = 2:2 (*w*/*v*), 150 rpm
		A1C3	Avicel:Cellulose = 1:3 (*w*/*v*), 150 rpm
		A0C4	Avicel:Cellulose = 0:4 (*w*/*v*), 150 rpm
	Agitation speed ^(2)^	150 rpm	Fixed C source ratio (A3C1), 150 rpm
		180 rpm	Fixed C source ratio (A3C1), 180 rpm
		210 rpm	Fixed C source ratio (A3C1), 210 rpm
	Turbulence efficiency ^(3)^	Baf-w/o BC	Fixed C source ratio (A3C1), 180 rpm, baffled flask, no biochar
		Baf-1% BC	Fixed C source ratio (A3C1), 180 rpm, baffled flask, 1% biochar
		Rou-w/o BC	Fixed C source ratio (A3C1), 180 rpm, round flask, no biochar
		Rou-1% BC	Fixed C source ratio (A3C1), 180 rpm, round flask, 1% biochar
Reactor		C0-150 (Control)	Avicel:Cellulose = 4:0 (*w*/*v*, control), 150 rpm, no turbulence
		A3-180	Fixed C source ratio (A3C1), 180 rpm, no turbulence
		A3-180_Imp	Fixed C source ratio (A3C1), 180 rpm, impeller adjusted (turbulence added)
		A3-210_Imp	Fixed C source ratio (A3C1), 210 rpm, impeller adjusted (turbulence added)

^(1)^ C source composition experiments were conducted at a fixed agitation speed of 150 rpm, with a total carbon concentration of 2% (*w*/*v*) and 1% (*w*/*v*) biochar. ^(2)^ Agitation speed experiments varied the shaking speed, while carbon source (A3C1), biochar, and other parameters were fixed. ^(3)^ Turbulence efficiency experiments were conducted using baffled and round-bottom flasks, Carbon source ration (A3C1) and agitation speed (180 rpm) were fised. Conditions included with or without 1% (*w*/*v*) biochar.

## Data Availability

The original contributions presented in this study are included in the article/Supplementary Materials. Further inquiries can be directed to the corresponding author.

## Supplementary Materials

The following supporting information can be downloaded at: https://www.mdpi.com/article/10.3390/ijms26083731/s1.

## References

[1] Razzak S.A., Hossain M.M., Lucky R.A., Bassi A.S., De Lasa H. (2013). Integrated CO_2_ capture, wastewater treatment and biofuel production by microalgae culturing—A review. Renew. Sust. Energy Rev..

[2] Ibrahim N.I., Shahar F.S., Hameed Sultan M.T., Md Shah A.U., Azrie Safri S.N., Mat Yazik M.H. (2021). Overview of bioplastic introduction and its applications in product packaging. Coatings.

[3] Rodionova M.V., Bozieva A.M., Zharmukhamedov S.K., Leong Y.K., Chi-Wei Lan J., Veziroglu A., Veziroglu T.N., Tomo T., Chang J.S., Allakhverdiev S.I. (2022). A comprehensive review on lignocellulosic biomass biorefinery for sustainable biofuel production. Int. J. Hydrogen Energy.

[4] Mangal M., Rao C.V., Banerjee T. (2023). Bioplastic: An eco-friendly alternative to non-biodegradable plastic. Polym. Int..

[5] Kudakasseril Kurian J., Raveendran Nair G., Hussain A., Vijaya Raghavan G.S. (2013). Feedstocks, logistics and pre-treatment processes for sustainable lignocellulosic biorefineries: A comprehensive review. Renew. Sust. Energy Rev..

[6] Rodionova M.V., Poudyal R.S., Tiwari I., Voloshin R.A., Zharmukhamedov S.K., Nam H.G., Zayadan B.K., Bruce B.D., Hou H.J.M., Allakhverdiev S.I. (2017). Biofuel production: Challenges and opportunities. Int. J. Hydrogen Energy.

[7] Yu D., Guo J., Meng J., Sun T. (2023). Biofuel production by hydro-thermal liquefaction of municipal solid waste: Process characterization and optimization. Chemosphere.

[8] Shah M., Rajhans M., Pandya H.A., Mankad A.U. (2021). Mankad bioplastic for future: A review Then and Now. World J. Adv. Res. Rev..

[9] Siqueira J.G.W., Rodrigues C., Vandenberghe L.P.d.S., Woiciechowski A.L., Soccol C.R. (2020). Current advances in on-site cellulase production and application on lignocellulosic biomass conversion to biofuels: A review. Biomass Bioenergy.

[10] REN21 Secretariat (2018). Renewable 2018 Global Status Report.

[11] Eckert S., Karassin O., Steinebach Y. (2024). A policy portfolio approach to plastics throughout their life cycle: Supranational and national regulation in the European Union. Environ. Policy Gov..

[12] Van Dyk J.S., Pletschke B.I. (2012). A review of lignocellulose bioconversion using enzymatic hydrolysis and synergistic cooperation between enzymes—Factors affecting enzymes, conversion and synergy. Biotechnol. Adv..

[13] Woo W.X., Tan J.P., Wu T.Y., Yeap S.K., Luthfi A.A.I., Manaf S.F.A., Jamali N.S., Hui Y.W. (2024). An overview on the factors affecting enzymatic saccharification of lignocellulosic biomass into fermentable sugars. Rev. Chem. Eng..

[14] Abo B.O., Gao M., Wang Y., Wu C., Ma H., Wang Q. (2019). Lignocellulosic biomass for bioethanol: An overview on pretreatment, hydrolysis and fermentation processes. Rev. Environ. Health.

[15] El-Zawawy W.K., Ibrahim M.M., Abdel-Fattah Y.R., Soliman N.A., Mahmoud M.M. (2011). Acid and enzyme hydrolysis to convert pretreated lignocellulosic materials into glucose for ethanol production. Carbohydr. Polym..

[16] Sukumaran R.K., Singhania R.R., Mathew G.M., Pandey A. (2009). Cellulase production using biomass feed stock and its application in lignocellulose saccharification for bio-ethanol production. Renew. Energy.

[17] Singh A., Bajar S., Devi A., Pant D. (2021). An overview on the recent developments in fungal cellulase production and their industrial applications. Bioresour. Technol. Rep..

[18] Klein-Marcuschamer D., Oleskowicz-Popiel P., Simmons B.A., Blanch H.W. (2012). The challenge of enzyme cost in the production of lignocellulosic biofuels. Biotechnol. Bioeng..

[19] Singh A., Bajar S., Devi A., Bishnoi N.R. (2021). Evaluation of cellulase production from *Aspergillus niger* and *Aspergillus heteromorphus* under submerged and solid-state fermentation. Environ. Sustain..

[20] Korsa G., Konwarh R., Masi C., Ayele A., Haile S. (2023). Microbial cellulase production and its potential application for textile industries. Ann. Microbiol..

[21] Srivastava N., Srivastava M., Mishra P.K., Singh P., Ramteke P.W. (2015). Application of cellulases in biofuels industries: An overview. J. Biofuels Bioenergy.

[22] Infanzón-Rodríguez M.I., Ragazzo-Sánchez J.A., del Moral S., Calderón-Santoyo M., Gutiérrez-Rivera B., Aguilar-Uscanga M.G. (2020). Optimization of cellulase production by *Aspergillus niger* ITV 02 from sweet sorghum bagasse in submerged culture using a Box–Behnken design. Sugar Tech..

[23] Matkar K., Chapla D., Divecha J., Nighojkar A., Madamwar D. (2013). Production of cellulase by a newly isolated strain of Aspergillus sydowii and its optimization under submerged fermentation. Int. Biodeterior. Biodegrad..

[24] Ramanathan G., Banupriya S., Abirami D. (2010). Production and optimization of cellulase from *Fusarium oxysporum* by submerged fermentation. Bioprocess. Biosyst. Eng..

[25] Reihani S.F.S., Khosravi-Darani K. (2019). Influencing factors on single-cell protein production by submerged fermentation: A review. Electron. J. Biotechnol..

[26] Cunha F.M., Esperança M.N., Zangirolami T.C., Badino A.C., Farinas C.S. (2012). Sequential solid-state and submerged cultivation of *Aspergillus niger* on sugarcane bagasse for the production of cellulase. Bioresour. Technol..

[27] Intasit R., Cheirsilp B., Suyotha W., Boonsawang P. (2021). Synergistic production of highly active enzymatic cocktails from lignocellulosic palm wastes by sequential solid-state submerged fermentation and co-cultivation of different filamentous fungi. Biochem. Eng. J..

[28] da Silva Delabona P., Lima D.J., Robl D., Rabelo S.C., Farinas C.S., da Cruz Pradella J.G. (2016). Enhanced cellulase production by *Trichoderma harzianum* by cultivation on glycerol followed by induction on cellulosic substrates. J. Ind. Microbiol. Biotechnol..

[29] Lan T.Q., Wei D., Yang S.T., Liu X. (2013). Enhanced cellulase production by Trichoderma viride in a rotating fibrous bed bioreactor. Bioresour. Technol..

[30] Myeong S., Yun J. (2024). Culture of *Trichoderma sp.* with biochar to produce high-activity cellulase in a laboratory. Bioresources.

[31] Du J., Zhang Y., Qu M., Yin Y., Fan K., Hu B., Zhang H., Wei M., Ma C. (2019). Effects of biochar on the microbial activity and community structure during sewage sludge composting. Bioresour. Technol..

[32] Awad Y.M., Lee S.S., Kim K.H., Ok Y.S., Kuzyakov Y. (2018). Carbon and nitrogen mineralization and enzyme activities in soil aggregate-size classes: Effects of biochar, oyster shells, and polymers. Chemosphere.

[33] Yin Y., Li M., Tao X., Yang C., Zhang W., Li H., Zheng Y., Wang X., Chen R. (2023). Biochar enhanced organic matter transformation during pig manure composting: Roles of the cellulase activity and fungal community. J. Environ. Manag..

[34] Mohanty S.K., Valenca R., Berger A.W., Yu I.K.M., Xiong X., Saunders T.M., Tsang D.C.W. (2018). Plenty of room for carbon on the ground: Potential applications of biochar for stormwater treatment. Sci. Total Environ..

[35] Wang J., Wang S. (2019). Preparation, modification and environmental application of biochar: A review. J. Clean. Prod..

[36] Zhang Y., Wang J., Feng Y. (2021). The effects of biochar addition on soil physicochemical properties: A review. Catena.

[37] Kim Y., Park S., Kim Y. (2020). Novel Trichoderma sp. KMF006 Strain Producing Cellulase with High Activity. Korean Patent.

[38] Kogo T., Yoshida Y., Koganei K., Matsumoto H., Watanabe T., Ogihara J., Kasumi T. (2017). Production of rice straw hydrolysis enzymes by the fungi *Trichoderma reesei* and *Humicola insolens* using rice straw as a carbon source. Bioresour. Technol..

[39] Silva J.C.R., Salgado J.C.S., Vici A.C., Ward R.J., Polizeli M.L.T.M., Guimarães L.H.S., Furriel R.P.M., Jorge J.A. (2020). A novel *Trichoderma reesei* mutant RP698 with enhanced cellulase production. Braz. J. Microbiol..

[40] Hodrová B., Kopečný J., Káš J. (1998). Cellulolytic enzymes of rumen anaerobic fungi *Orpinomyces joyonii* and *Caecomyces communis*. Res. Microbiol..

[41] Karnchanatat A., Petsom A., Sangvanich P., Piapukiew J., Whalley A.J.S., Reynolds C.D., Gadd G.M., Sihanonth P. (2008). A novel thermostable endoglucanase from the wood-decaying fungus *Daldinia eschscholzii* (Ehrenb.:Fr.) Rehm. Enzyme Microb. Technol..

[42] Singhania R.R., Saini R., Adsul M., Saini J.K., Mathur A., Tuli D. (2015). An integrative process for bio-ethanol production employing SSF produced cellulase without extraction. Biochem. Eng. J..

[43] Saini R., Saini J.K., Adsul M., Patel A.K., Mathur A., Tuli D., Singhania R.R. (2015). Enhanced cellulase production by *Penicillium oxalicum* for bio-ethanol application. Bioresour. Technol..

[44] Gao J., Qian Y., Wang Y., Qu Y., Zhong Y. (2017). Production of the versatile cellulase for cellulose bioconversion and cellulase inducer synthesis by genetic improvement of *Trichoderma reesei*. Biotechnol. Biofuels.

[45] Li Y., Liu C., Bai F., Zhao X. (2016). Overproduction of cellulase by *Trichoderma reesei* RUT C30 through batch-feeding of synthesized low-cost sugar mixture. Bioresour. Technol..

[46] Suto M., Tomita F. (2001). Induction and catabolite repression mechanisms of cellulase in fungi. J. Biosci. Bioeng..

[47] Kubicek C.P., Mikus M., Schuster A., Schmoll M., Seiboth B. (2009). Metabolic engineering strategies for the improvement of cellulase production by *Hypocrea jecorina*. Biotechnol. Biofuels.

[48] Yu L., Chao Y., Wensel P., Chen S. (2012). Hydrodynamic and kinetic study of cellulase production by *Trichoderma reesei* with pellet morphology. Biotechnol. Bioeng..

[49] Sirohi R., Singh A., Tarafdar A., Shahi N.C., Verma A.K., Kushwaha A. (2019). Cellulase production from pre-treated pea hulls using *Trichoderma reesei* under submerged fermentation. Waste Biomass Valorization.

[50] Teoh Y., Don M.M., Fadzilah K. (2017). Optimization of cellulase production by *Pycnoporus sanguineus* in 5 L stirred tank bioreactor and enhanced fermentation by employing external loop. Chiang Mai J. Sci..

[51] Mmango-Kaseke Z., Okaiyeto K., Nwodo U.U., Mabinya L.V., Okoh A.I. (2016). Optimization of cellulase and xylanase production by *Micrococcus* species under submerged fermentation. Sustainability.

[52] Park J.K., Hyun S.H., Jung J.Y. (2004). Conversion of *G. hansenii* PJK into non-cellulose-producing mutants according to the culture condition. Biotechnol. Bioprocess. Eng..

[53] Mitra S., Banerjee P., Gachhui R., Mukherjee J. (2011). Cellulase and xylanase activity in relation to biofilm formation by two intertidal filamentous fungi in a novel polymethylmethacrylate conico-cylindrical flask. Bioprocess. Biosyst. Eng..

[54] Batra J., Beri D., Mishra S. (2014). Response surface methodology-based optimization of β-glucosidase production from *Pichia pastoris*. Appl. Biochem. Biotechnol..

[55] Weber J., Agblevor F.A. (2005). Microbubble fermentation of *Trichoderma reesei* for cellulase production. Process Biochem..

[56] Ma H., Liu Y., Li Z. (2023). The synergistic hydrogen production of bicellular fermentation systems and fluid dynamics simulation in reactor under stirring. Bioresour. Technol. Rep..

[57] Dey P., Rangarajan V., Singh J., Nayak J., Dilip K.J. (2022). Current perspective on improved fermentative production and purification of fungal cellulases for successful biorefinery applications: A brief review. Biomass Convers. Biorefin..

[58] Shokrkar H., Ebrahimi S., Zamani M. (2018). A review of bioreactor technology used for enzymatic hydrolysis of cellulosic materials. Cellulose.

[59] Chisti Y., Jauregui-Haza U.J. (2002). Oxygen transfer and mixing in mechanically agitated airlift bioreactors. Bioprocess. Eng..

[60] de Albuquerque de Carvalho M.L., Carvalho D.F., de Barros Gomes E., Maeda R.N., Anna L.M.M.S., de Castro A.M., Pereira N. (2014). Optimisation of cellulase production by *Penicillium funiculosum* in a stirred tank bioreactor using multivariate response surface analysis. Enzyme Res..

[61] Kim S.W., Kang S.W., Lee J.S. (1997). Cellulase and xylanase production by *Aspergillus niger* KKS in various bioreactors. Bioresour. Technol..

[62] Ahamed A., Vermette P. (2008). Culture-based strategies to enhance cellulase enzyme production from *Trichoderma reesei* RUT-C30 in bioreactor culture conditions. Biochem. Eng. J..

[63] Lejeune R., Baron G.V. (1995). Effect of agitation on growth and enzyme production of *Trichoderma reesei* in batch fermentation. Biotechnol. Bioeng..

[64] Hayward T.K., Hamilton J., Tholudur A., McMillan J.D. (2000). Improvements in titer, productivity, and yield using Solka-Floc for cellulase production. Proceedings of the Twenty-First Symposium on Biotechnology for Fuels and Chemicals.

[65] Nelson N. (1944). A photometric adaptation of the Somogyi method for the determination of glucose. J. Biol. Chem..

[66] Joo A.R., Jeya M., Lee K.M., Sim W.I., Kim J.S., Kim I.W., Kim Y.S., Oh D.K., Gunasekaran P., Lee J.K. (2009). Purification and characterization of a β-1,4-glucosidase from a newly isolated strain of *Fomitopsis pinicola*. Appl. Microbiol. Biotechnol..

[67] Bradford M.M. (1976). A rapid and sensitive method for the quantitation of microgram quantities of protein utilizing the principle of protein-dye binding. Anal. Biochem..

[68] NREL (2008). Measurement of cellulase activities: Laboratory analytical procedure (LAP). NREL Technical Report (NREL/TP-510-42628).

