# Supplementary material for: Optimization and Bioreactor Scale-Up of Cellulase Production in Trichoderma sp. KMF006 for Higher Yield and Performance
Source: Int J Mol Sci. 2025 Apr 15;26(8):3731. doi: 10.3390/ijms26083731 (PMC12027645; doi:10.3390/ijms26083731)
Supplement: Supplementary file 1 [file ijms-26-03731-s001.zip › ijms-3568452-supplementary.pdf]

[Supplementary Materials]

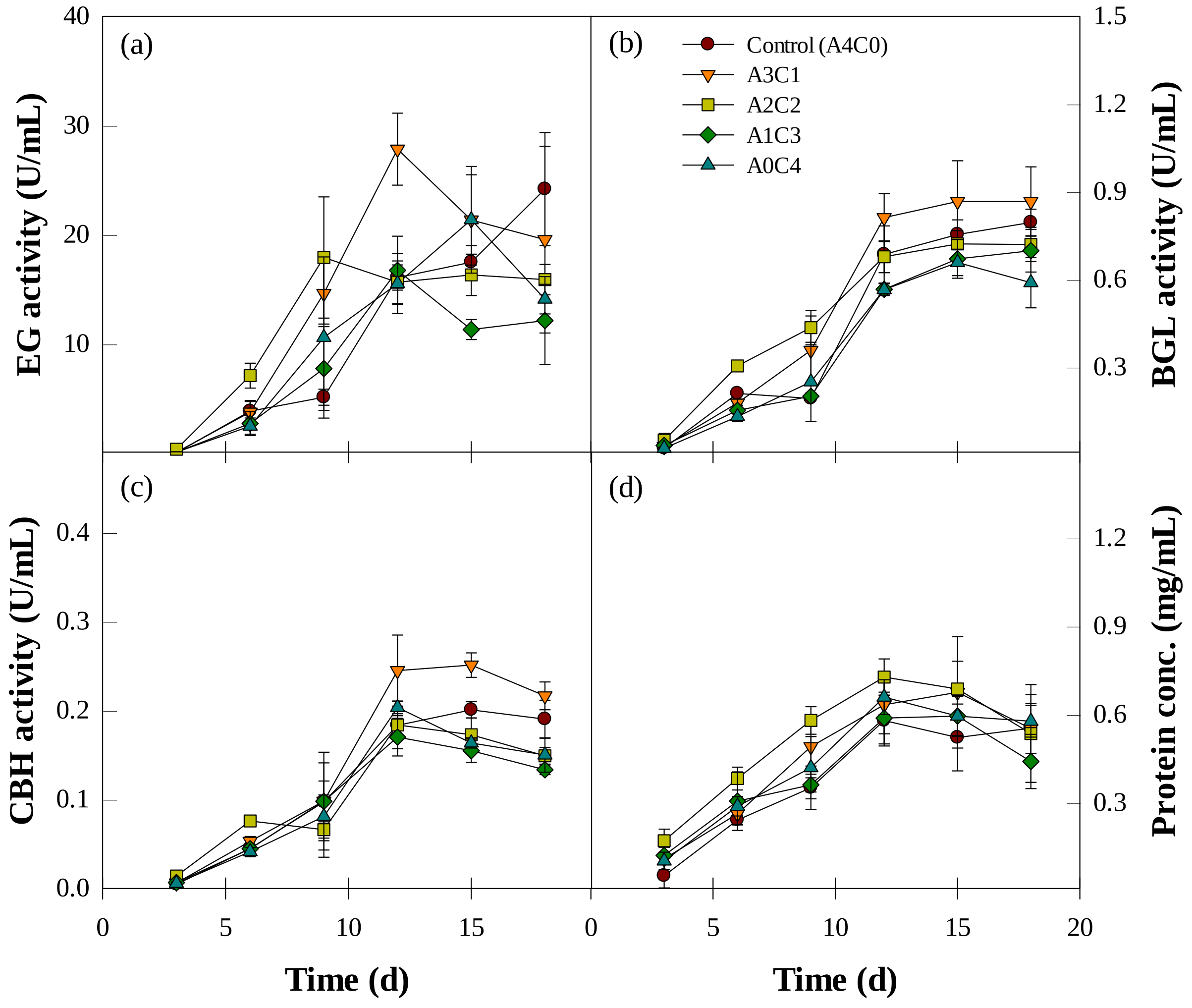

Fig. S1. Time profiles of cellulase activities (EG, BGL, and CBH) and protein concentration of KMF006 cultured under different carbon source compositions in flask-scale cultures. (a) EG (endoglucanase) activity, (b) BGL (β-glucosidase) activity, (c) CBH (cellobiohydrolase) activity, and (d) protein concentration. The experimental conditions include A4C0 (Avicel:Cellulose = 4:0, control); A3C1 (Avicel:Cellulose = 3:1); A2C2 (Avicel:Cellulose = 2:2); A1C3 (Avicel:Cellulose = 1:3); and A0C4 (Avicel:Cellulose = 0:4). Error bars represent standard deviation (SD) from six measurements (*n*=6).

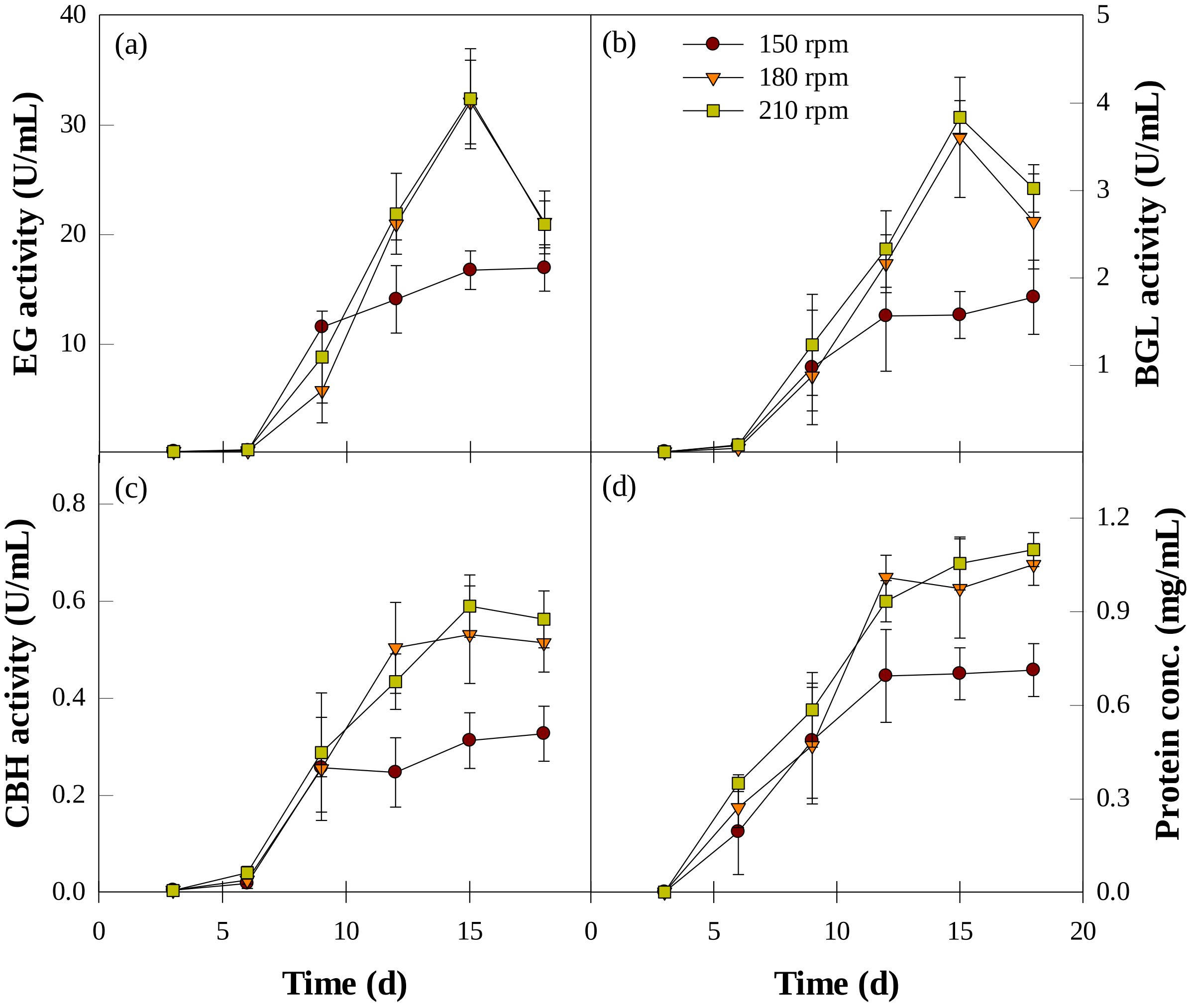

Fig. S2. Time profiles of cellulase activities (EG, BGL, and CBH) and protein concentration at different agitation speeds (150, 180, and 210 rpm) in flask-scale cultures. (a) EG (endoglucanase) activity, (b) BGL (β-glucosidase) activity, (c) CBH (cellobiohydrolase) activity, and (d) protein concentration. Error bars represent standard deviation (SD) from six measurements (*n*=6).

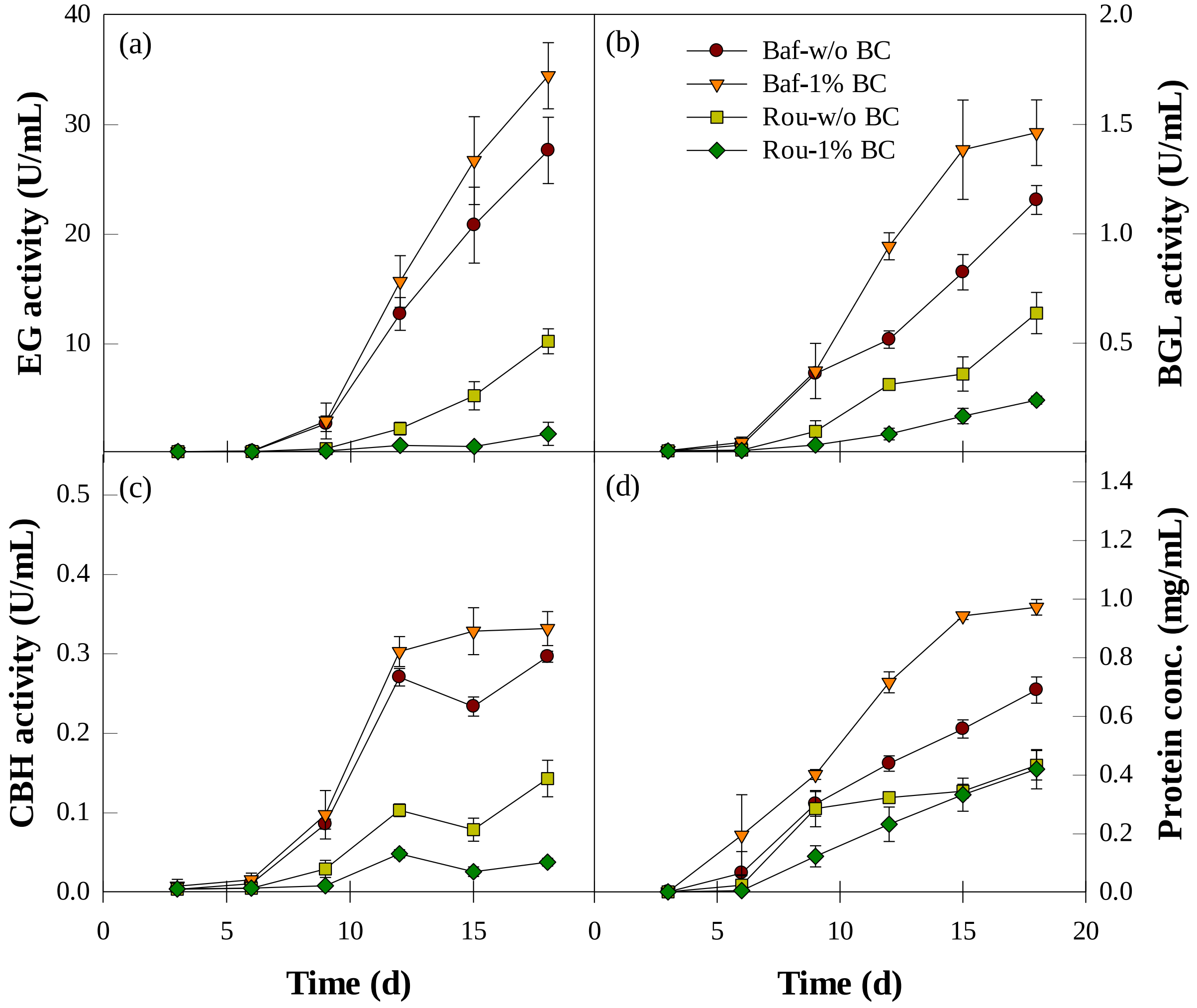

Fig. S3. Time profiles of cellulase activities (EG, BGL, and CBH) and protein concentration under different turbulence efficiencies in flask scale. (a) EG (endoglucanase) activity, (b) BGL (β-glucosidase) activity, (c) CBH (cellobiohydrolase) activity, and (d) protein concentration. Experimental conditions are represented as follows: **Baf-w/o BC**, Baffled flask without biochar; **Baf-1% BC**, Baffled flask with 1% biochar; **Rou-w/o BC**, Round flask without biochar; **Rou-1% BC**, Round flask with 1% biochar. Error bars represent standard deviation (SD) from six measurements (*n*=6).

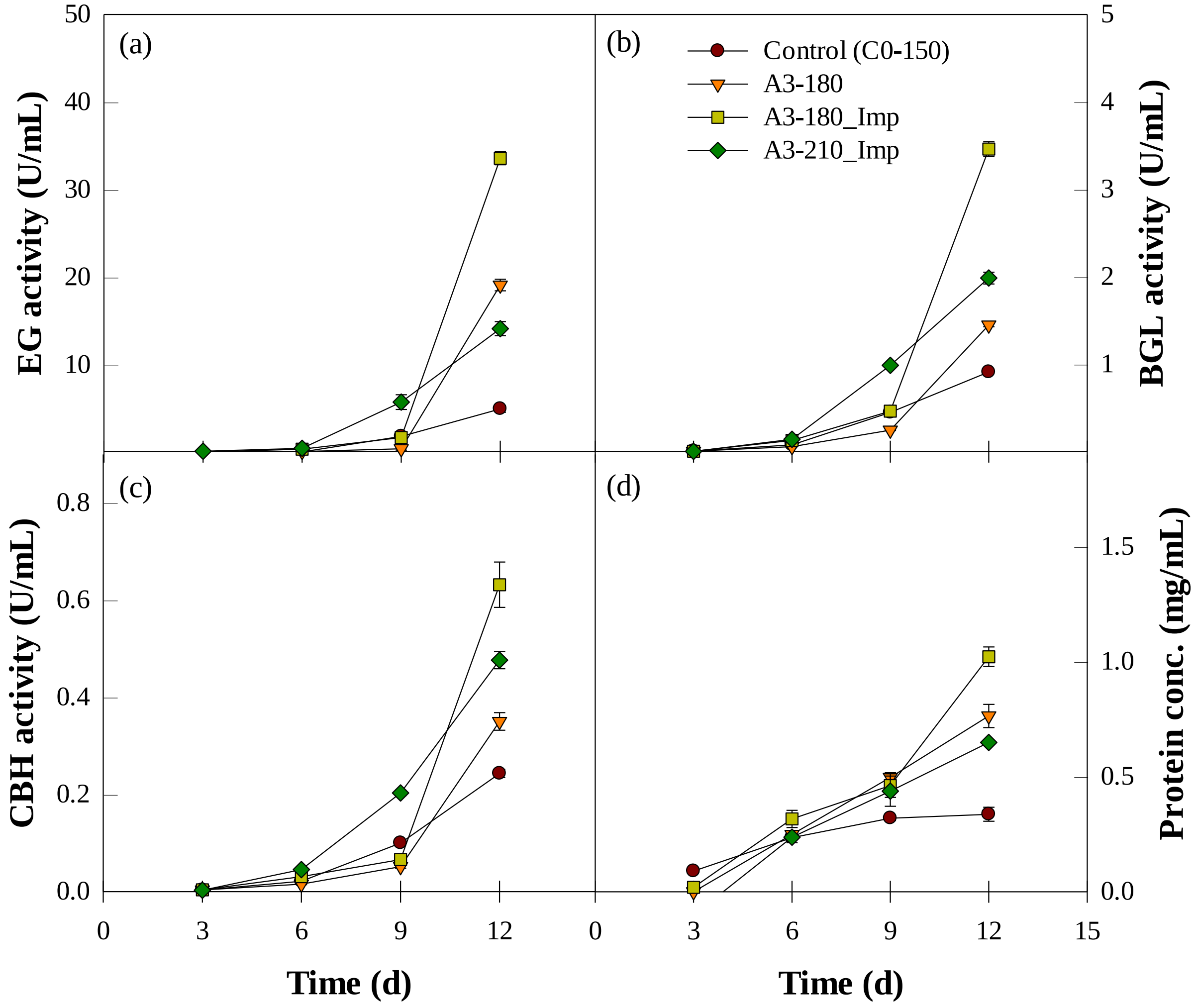

Fig. S4. Time profiles of cellulase activities (EG, BGL, and CBH) and protein concentration of KMF006 cultured with 1% biochar in a 10 L submerged fermentation (SmF) reactor. (a) EG (endoglucanase) activity, (b) BGL (β-glucosidase) activity, (c) CBH (cellobiohydrolase) activity, and (d) protein concentration. Experimental conditions are represented as follows: **C0-150**, Control condition (Avicel:Cellulose = 4:1, 150 rpm); **A3-180**, Avicel:Cellulose = 3:1, 180 rpm; **A3-180_Imp**, Avicel:Cellulose = 3:1, 180 rpm with impeller adjustment; **A3-210_Imp**, Avicel:Cellulose = 3:1, 210 rpm with impeller adjustment. Error bars represent standard deviation (SD) from three biological replicates measured in duplicate (*n*=6).
